# Case Report: Transient Complete Atrioventricular Block During Coronary Sinus Reducer Implantation: An Unexpected Complication

**DOI:** 10.3390/reports9030197

**Published:** 2026-06-23

**Authors:** Gianluca Pagnoni, Alberto Monello, Luca Rossi, Daniela Aschieri, Marco Loffi

**Affiliations:** Cardiology Unit of Emergency Department, Guglielmo da Saliceto Hospital, 29121 Piacenza, Italy; a.monello@ausl.pc.it (A.M.); dr.luca.rossi.20@gmail.com (L.R.);

**Keywords:** refractory angina, Coronary Sinus Reducer, complete atrioventricular block, temporary pacing

## Abstract

**Background and Clinical Significance:** The Coronary Sinus Reducer (CSR) is a percutaneous therapeutic option for patients with refractory angina who are unsuitable for further myocardial revascularization. The procedure has a generally favorable safety profile, with a low rate of reported procedural complications. To our knowledge, major atrioventricular (AV) conduction disturbances during CSR implantation have not been previously described. This case highlights a rare but clinically relevant intraprocedural complication; **Case Presentation:** A 71-year-old man with multivessel coronary artery disease and previous coronary artery bypass grafting was referred for CSR implantation because of refractory angina despite optimal medical therapy and lack of further revascularization options. The procedure was performed via a right jugular venous approach. Baseline electrocardiography showed right bundle branch block and findings consistent with previous inferior myocardial infarction, without definite criteria for left anterior fascicular block. During coronary sinus cannulation, the patient developed transient complete AV block, resulting in an approximately 8–10-second ventricular pause without a stable ventricular escape rhythm. The conduction disturbance resolved after catheter withdrawal and repositioning. Given the severity of the event, a temporary transvenous pacemaker was inserted via the right femoral vein, allowing safe completion of CSR implantation. At three-month follow-up, angina had improved from Canadian Cardiovascular Society class III to class I, and no recurrent advanced AV block was documented; **Conclusions:** Transient complete AV block may occur during CSR implantation, particularly during coronary sinus manipulation and possibly in patients with pre-existing conduction disease. Careful catheter handling, prompt recognition of conduction disturbances, and immediate availability of temporary pacing support should be considered in selected high-risk patients undergoing CSR implantation.

## 1. Introduction and Clinical Significance

The Coronary Sinus Reducer (CSR) is a stent-shaped device with a central narrowing that, once implanted in the coronary sinus (CS), creates a calibrated narrowing, increasing coronary venous pressure and favoring redistribution of flow with improved subendocardial perfusion in ischemic territories not amenable to myocardial revascularization [1,2].

Refractory angina remains a challenging clinical condition in patients with advanced coronary artery disease who continue to experience symptoms despite optimal medical therapy and who have no further revascularization options. In this setting, CSR implantation has emerged as a therapeutic strategy aimed primarily at improving symptoms and quality of life rather than modifying epicardial coronary anatomy. As the number of patients treated with CSR increases, recognition of uncommon procedural complications becomes clinically relevant, particularly in subjects with complex venous anatomy or pre-existing conduction abnormalities.

CSR is indicated for refractory angina in patients who are not candidates for revascularization and has shown significant clinical benefits both in the randomized COSIRA trial and in subsequent multicenter registries, confirming a favorable efficacy and safety profile [3,4,5]. Major complications are rare (2–3%) and include device thrombosis, displacement/embolization, coronary sinus dissection or perforation, and periprocedural myocardial infarction.

To the best of our knowledge, complete AV block occurring during CSR implantation itself has not been previously reported in the literature.

The close anatomic relationship between the CS ostium, Koch’s triangle, the atrioventricular (AV) node, and the His bundle makes mechanical or irritative interference with the conduction system a plausible, albeit infrequent, mechanism during CS cannulation maneuvers. Similar risks are recognized in procedures that require CS manipulation (e.g., CRT lead placement) or direct interaction with the conduction axis (His bundle pacing). This suggests that AV block might occasionally occur during CSR, especially in the presence of anatomic variants or pre-existing conduction disease [6,7].

Based on these considerations, we report a case of transient complete AV block occurring during CSR implantation in a patient with baseline right bundle branch block and findings consistent with previous inferior myocardial infarction, without definite criteria for left anterior fascicular block.

## 2. Case Presentation

A 71-year-old man with triple-vessel coronary artery disease had undergone triple coronary artery bypass grafting in 2010, followed by percutaneous coronary intervention on the left anterior descending (LAD) artery in 2019 for occlusion of an arterial graft.

Past medical history included hypertension and dyslipidemia with statin intolerance. Anti-ischemic and antianginal therapy was optimized (aspirin, clopidogrel, β-blocker, angiotensin receptor blocker, ranolazine, ezetimibe, PCSK9 inhibitor, transdermal nitroglycerin). He presented to the emergency department with exertional angina for approximately one year and recent worsening. On arrival he was asymptomatic at rest with stable vital signs (BP 150/80 mmHg, HR 70 bpm, SpO_2_ 98%). The electrocardiogram showed sinus rhythm with right bundle branch block and findings consistent with previous inferior myocardial infarction, without definite criteria for left anterior fascicular block. Echocardiography documented a left ventricular ejection fraction of 45%. This mildly reduced left ventricular ejection fraction was already known from previous evaluations and did not represent a new finding at the time of admission. Right ventricular systolic function was preserved, with TAPSE of 21 mm and tissue Doppler-derived tricuspid annular systolic velocity (TDI S′) of 10 cm/s. These indices are commonly used in routine echocardiographic assessment, with TDI S′ showing favorable reproducibility compared with TAPSE [8]. High-sensitivity troponin I values were 45–49 ng/L, above the local 99th percentile upper reference limit for men (<20 ng/L), without a significant rise-and-fall pattern on serial testing. In the absence of acute ischemic ECG changes or dynamic troponin variation, he was admitted with a diagnosis of stable angina, Canadian Cardiovascular Society (CCS) class III (Figure 1).

Coronary angiography revealed multivessel coronary disease with chronic total occlusion of an obtuse marginal (OM) branch, a hypoplastic right coronary artery with a proximal stenosis not suitable for revascularization, good prior stent result on the LAD, and occluded bypass grafts. A similar picture had been documented in 2024.

Given the lack of revascularization targets, CSR implantation via the right internal jugular vein was planned. Coronary sinus cannulation was performed using a 9-Fr guiding catheter/delivery system compatible with Coronary Sinus Reducer implantation and a Multipurpose catheter. The coronary sinus was accessed by advancing the guiding catheter within the right atrium under gentle counterclockwise rotation, without intentional advancement into the right ventricle.

Before coronary sinus cannulation, the ECG showed sinus rhythm with right bundle branch block and findings consistent with previous inferior myocardial infarction, without definite criteria for left anterior fascicular block. During manipulation of the Multipurpose catheter at the coronary sinus ostium, transient complete AV block occurred, likely due to mechanical interaction with the AV node–His bundle region. The episode lasted approximately 8–10 s and was characterized by a ventricular pause/asystolic segment on the procedural monitor, without a stable ventricular escape rhythm (Figure 2). At the time of the event, the patient remained conscious and hemodynamically stable, without chest pain or loss of consciousness; no atropine, isoproterenol, or other chronotropic medications were administered. The rhythm recovered spontaneously after catheter withdrawal/repositioning.

Because of the need for further coronary sinus manipulation in a tortuous venous anatomy and the possibility of recurrence, a temporary transvenous pacing lead was inserted via the right femoral vein and positioned in the right ventricle. The temporary pacemaker was maintained as procedural and early post-procedural backup and was removed after the observation period once no recurrent high-grade AV block, significant pauses, or pacing-dependent episodes had been documented. Device deployment was then completed successfully despite marked tortuosity of the coronary sinus (Figure 3).

During the 72 h in-hospital observation period, continuous telemetry did not show recurrent high-grade AV block, significant pauses, or pacing-dependent episodes. A 12-lead ECG obtained before discharge showed sinus rhythm with persistence of the pre-existing right bundle branch block and findings consistent with previous inferior myocardial infarction, without new PR prolongation or additional conduction abnormalities. The patient was therefore discharged without indication for permanent pacemaker implantation. At the three-month outpatient follow-up, he reported CCS class I angina. Repeat 12-lead ECG showed an unchanged conduction pattern compared with baseline, and 24 h ambulatory rhythm monitoring did not document recurrent advanced AV block, clinically significant pauses, or progression of conduction disease.

Beyond procedural success and symptom relief, continued optimization of secondary prevention and individualized exercise-based rehabilitation should be encouraged, as adherence to rehabilitation programs remains a relevant challenge in patients with cardiovascular disease [9].

## 3. Discussion

This case shows transient advanced AV block arising during coronary sinus cannulation, most likely from mechanical irritation of the AV node–His bundle region. Despite the generally low rate of procedural complications, the contiguity of the CS ostium, Koch’s triangle, the AV node, and the His bundle provides a plausible explanation for rare AV block events in predisposed conditions, as already reported with other procedures that require CS cannulation (CRT, His bundle pacing) [6,7,10,11].

Comparison with previous reports. Published experience on the interaction between CSR and cardiac pacing or resynchronization systems remains limited. Farooqui et al. reported successful CSR implantation in a patient with a pre-existing left ventricular lead, emphasizing the feasibility of the procedure but also the potential concern that the lead could be trapped between the CSR device and the coronary sinus wall, complicating future extraction or repositioning [10]. Mrak et al. described successful cardiac resynchronization using His bundle pacing in a patient after previous CSR implantation, highlighting that a reducer positioned in the coronary sinus may limit conventional left ventricular lead placement and that conduction-system pacing may represent an alternative strategy [11]. In contrast to these reports, our case did not involve a pre-existing or subsequent resynchronization system, but describes an acute transient complete AV block occurring during CSR implantation itself. This distinction supports the novelty of the present case and suggests that, beyond device–lead interactions, direct mechanical irritation during CS cannulation may represent an additional procedural risk in selected patients.

The available literature on AV conduction disturbances during coronary sinus lead implantation is limited and mainly derives from CRT procedures and technically challenging left ventricular lead placement. In CRT implantation, selective cannulation of the coronary sinus and its tributaries is mandatory, but it may require repeated catheter, sheath, guidewire, or lead manipulation close to the CS ostium and posteroseptal right atrium, particularly in the presence of tortuous venous anatomy, unstable sheath support, or unfavorable target-vein angulation. In a series of 244 patients undergoing coronary sinus lead implantation for CRT, Azizi et al. reported perioperative complications in 12.5% of procedures, including coronary sinus dissection, perforation, ventricular fibrillation, and asystole, and emphasized that immediate defibrillation and pacing capability should be available during implantation [12,13]. Although transient AV block is rarely described as a specific complication, these observations support the concept that clinically relevant bradyarrhythmic events may occur during coronary sinus instrumentation. Anatomical studies further support this mechanism, as the CS ostium lies close to Koch’s triangle, the compact AV node, and the penetrating His bundle [6,7]. Therefore, temporary AV block during coronary sinus lead implantation is generally interpreted as a rare mechanical phenomenon caused by catheter- or sheath-induced irritation of the AV nodal–Hisian region, especially when manipulation is prolonged, forceful, or repeated. This experience from CRT procedures is relevant to CSR implantation because both procedures require instrumentation of the coronary sinus and may involve similar ostial/septal mechanical interactions. Similarly, His bundle pacing and electrophysiology mapping procedures have demonstrated that the proximity of the CS to the AV node and conduction system may result in transient block or even permanent injury to the atrioventricular conduction axis [7,11]. These experiences support the hypothesis that the complication observed in our case is a plausible consequence of catheter-induced mechanical stimulation.

The access technique used in the present case may also provide mechanistic insight. The coronary sinus was approached from the right atrium by gentle counterclockwise rotation of the guiding catheter, without intentional advancement of the catheter into the right ventricle. Therefore, the most plausible mechanism is transient mechanical stunning or irritation of the AV node–His bundle region from the right atrial/posteroseptal side, rather than injury to the left posterior fascicle within the right ventricle. This distinction is clinically relevant because His bundle irritation may potentially induce AV block even in patients without pre-existing conduction disease, whereas trauma to the left posterior fascicle would be expected to cause complete AV block mainly in patients with pre-existing bifascicular block.

A pre-procedural electrophysiological evaluation may also be considered in selected patients with significant baseline conduction abnormalities, although its routine use before CSR implantation cannot be recommended on the basis of the present case. Measurement of the HV interval could theoretically help identify patients with impaired His–Purkinje conduction reserve who may be at higher risk of developing high-grade AV block during coronary sinus manipulation. However, the clinical value, cost-effectiveness, and selection criteria for such an approach remain undefined and should be investigated in future studies.

Alternative cannulation strategies may also reduce mechanical interaction with the AV nodal–Hisian region in anatomically challenging cases. These include the use of catheters with different curves, softer or more supportive guiding systems, steerable sheaths, careful guidewire-assisted techniques, and imaging-guided approaches to minimize repeated or forceful manipulation at the coronary sinus ostium. In patients with suspected complex coronary venous anatomy, pre-procedural imaging of the coronary sinus, such as CT venography, may help define ostial orientation, venous tortuosity, and unfavorable anatomy before the procedure. This information could support procedural planning, guide catheter selection, and contribute to risk stratification, although dedicated evidence in the setting of CSR implantation is currently limited.

In our patient, baseline right bundle branch block reflected pre-existing intraventricular conduction disease, while marked coronary sinus tortuosity requiring repeated maneuvers likely amplified the risk of mechanical irritation of the AV node–His bundle region.

Practical implications. (i) Consider prophylactic temporary pacing in CSR candidates with significant baseline conduction abnormalities or anticipated difficult CS cannulation; (ii) minimize ostial/septal manipulation and limit repeated traumatic passes; (iii) ensure immediate pacing backup, prompt recognition of prolonged pauses, and post-procedure rhythm monitoring to detect delayed conduction disturbances. Although this event was not an out-of-hospital cardiac arrest, evidence from emergency cardiovascular systems emphasizes that avoidable delays in recognition and treatment are critical determinants of outcome; by analogy, CSR procedures in patients at higher conduction risk should include predefined rapid-response pathways and immediate pacing capability [14,15].

## 4. Conclusions

The Coronary Sinus Reducer (CSR) is a percutaneous therapeutic option for refractory angina in patients who are not candidates for revascularization. Major AV conduction disturbances have not been previously reported during CSR implantation. We report a 71-year-old man with non-revascularizable multivessel disease and baseline right bundle branch block, with findings consistent with previous inferior myocardial infarction but without definite electrocardiographic criteria for left anterior fascicular block. During CS cannulation, a transient complete AV block occurred, likely from mechanical irritation of the AV node–His axis in the setting of a tortuous CS anatomy; a temporary pacing system was placed, and implantation was completed without further complications. The subsequent course was uneventful, with symptomatic improvement at three months. This report underscores the need for careful CS manipulation, prompt recognition of conduction disturbances, and individualized procedural planning. In patients with significant baseline conduction abnormalities or suspected difficult coronary sinus anatomy, prophylactic temporary pacing may be considered before CSR implantation.

## Figures and Tables

**Figure 1:**
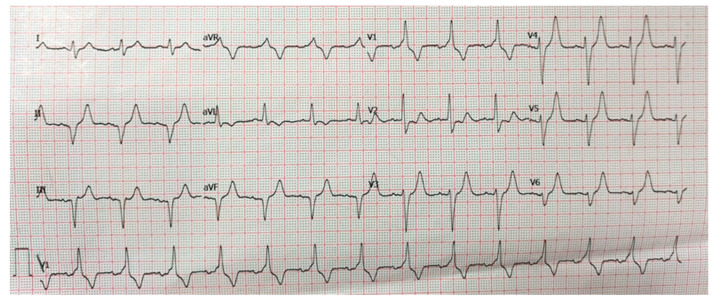
Baseline electrocardiogram showing sinus rhythm, right bundle branch block, and findings consistent with previous inferior myocardial infarction, without definite electrocardiographic criteria for left anterior fascicular block.

**Figure 2:**
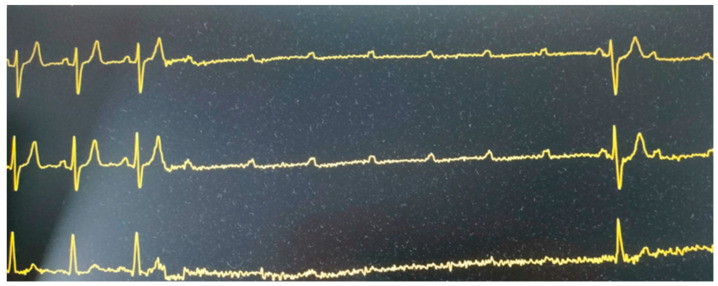
Procedural monitor during Coronary Sinus Reducer implantation showing transient complete atrioventricular block during coronary sinus cannulation, with an approximately 8–10 s ventricular pause/asystolic segment and no stable ventricular escape rhythm.

**Figure 3:**
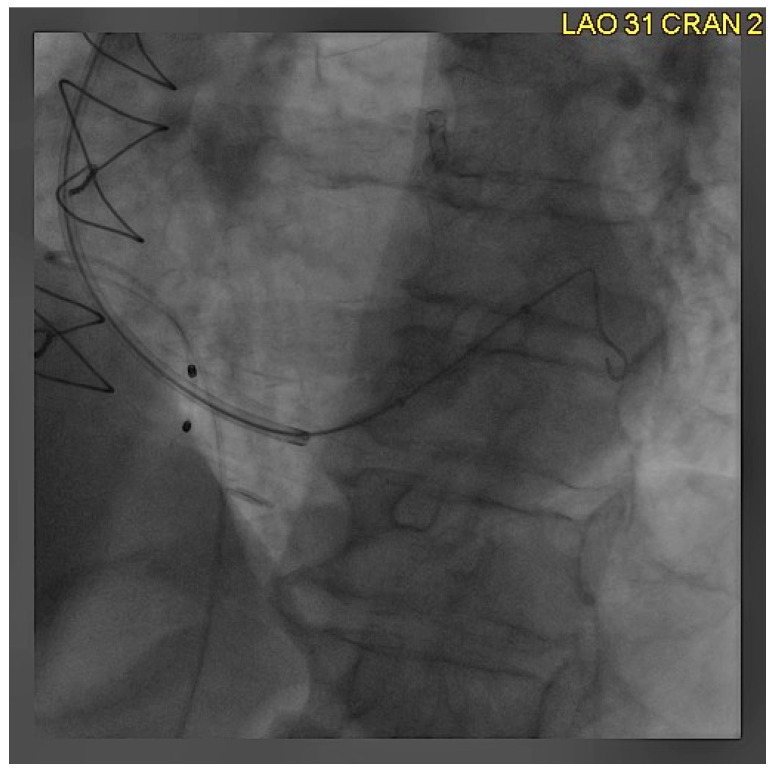
Fluoroscopy showing deployment of the Reducer device in the coronary sinus via right jugular access; radiopaque markers of the Reducer delivery balloon are visible. A temporary right ventricular pacing lead introduced from the right femoral vein is also visible.

## Data Availability

The data supporting the findings of this case report are available from the corresponding author upon reasonable request, in accordance with privacy and ethical restrictions.
